# Prognostic and risk factor analysis of cancer patients after unplanned ICU admission: a real-world multicenter study

**DOI:** 10.1038/s41598-023-49219-6

**Published:** 2023-12-15

**Authors:** Miao Wei, Mingguang Huang, Yan Duan, Donghao Wang, Xuezhong Xing, Rongxi Quan, Guoxing Zhang, Kaizhong Liu, Biao Zhu, Yong Ye, Dongmin Zhou, Jianghong Zhao, Gang Ma, Zhengying Jiang, Bing Huang, Shanling Xu, Yun Xiao, Linlin Zhang, Hongzhi Wang, Ruiyun Lin, Shuliang Ma, Yu’an Qiu, Changsong Wang, Zhen Zheng, Ni Sun, Lewu Xian, Ji Li, Ming Zhang, Zhijun Guo, Yong Tao, Li Zhang, Xiangzhe Zhou, Wei Chen, Daoxie Wang, Jiyan Chi

**Affiliations:** 1 Department of Intensive Care Unit, Shanxi Province Cancer Hospital/Shanxi Hospital Affiliated to Cancer Hospital, Chinese Academy of Medical Sciences/Cancer Hospital Affiliated to Shanxi Medical University, Taiyuan, Shanxi China; 2 Department of Intensive Care Unit, Key Laboratory of Cancer Prevention and Therapy, National Clinical Research Center of Cancer, Tianjin Medical University Cancer Institute and Hospital, Tianjin, China; 3 Department of Intensive Care Unit, Cancer Hospital Chinese Academy of Medical Sciences, Beijing, China; 4 Department of Intensive Care Unit, Cancer Hospital of Xinjiang Uygur Autonomous Region, Urumqi, Xinjiang China; 5 Department of Intensive Care Unit, Gaoxin District of Jilin Cancer Hospital, Changchun, Jilin China; 6 Department of Intensive Care Unit, Zhejiang Cancer Hospital, Hangzhou, Zhejiang China; 7 Department of Intensive Care Unit, Fudan University Affiliated Shanghai Cancer Hospital, Shanghai, China; 8 Department of Intensive Care Unit, Fujian Cancer Hospital and Fujian Medical University Cancer Hospital, Fuzhou, Fujian China; 9 Department of Intensive Care Unit, Henan Cancer Hospital, Zhengzhou, Henan China; 10 Department of Intensive Care Unit, Hunan Cancer Hospital, Changsha, Hunan China; 11 Department of Intensive Care Unit, Sun Yat-Sen University Cancer Center, Guangzhou, Guangdong China; 12 Department of Intensive Care Unit, Chongqing University Cancer Hospital, Chongqing, Sichuan China; 13 Department of Intensive Care Unit, Guangxi Medical University Affiliated Tumor Hospital, Nanning, Guangxi China; 14 Department of Intensive Care Unit, Sichuan Cancer Hospital and Institute, Chengdu, Sichuan China; 15 Department of Intensive Care Unit, Yunnan Cancer Hospital, Kunming, Yunnan China; 16 Department of Intensive Care Unit, Anhui Province Cancer Hospital, Hefei, Anhui China; 17 Department of Intensive Care Unit, Beijing Cancer Hospital, Beijing, China; 18 Department of Intensive Care Unit, Cancer Hospital of Shantou University Medical College, Shantou, Guangdong China; 19 Department of Intensive Care Unit, Jiangsu Cancer Hospital, Nanjing, Jiangsu China; 20 Department of Intensive Care Unit, Jiangxi Provincial Tumor Hospital, Nanchang, Jiangxi China; 21 Department of Intensive Care Unit, Harbin Medical University Cancer Hospital, Harbin, Heilongjiang China; 22 Department of Intensive Care Unit, Liaoning Cancer Hospital and Institute, Shenyang, Liaoning China; 23 Department of Intensive Care Unit, Huguang District of Jilin Cancer Hospital, Changchun, Jilin China; 24 Department of Intensive Care Unit, Affiliated Cancer Hospital and Institute of Guangzhou Medical University, Guangzhou, Guangdong China; 25 Department of Intensive Care Unit, Hainan Cancer Hospital, Haikou, Hainan China; 26 Department of Intensive Care Unit, Hangzhou Cancer Hospital, Hangzhou, Zhejiang China; 27 Department of Intensive Care Unit, Shandong First Medical University Affiliated Tumor Hospital, Jinan, Shandong China; 28 Department of Intensive Care Unit, Nantong Tumor Hospital, Nantong, Jiangsu China; 29 Department of Intensive Care Unit, Hubei Cancer Hospital, Wuhan, Hubei China; 30 Department of Intensive Care Unit, Gansu Provincial Cancer Hospital, Lanzhou, Gansu China; 31 Department of Intensive Care Unit, Beijing Shijitan Hospital (Capital Medical University Cancer Hospital), Beijing, China; 32 Department of Intensive Care Unit, Cancer Hospital of Zhengzhou, Zhengzhou, Henan China; 33 Department of Intensive Care Unit, Tumor Hospital of Mudanjiang City, Mudanjiang, Heilongjiang China

**Keywords:** Cancer, Oncology, Risk factors

## Abstract

To investigate the occurrence and 90-day mortality of cancer patients following unplanned admission to the intensive care unit (ICU), as well as to develop a risk prediction model for their 90-day prognosis. We prospectively analyzed data from cancer patients who were admitted to the ICU without prior planning within the past 7 days, specifically between May 12, 2021, and July 12, 2021. The patients were grouped based on their 90-day survival status, and the aim was to identify the risk factors influencing their survival status. A total of 1488 cases were included in the study, with an average age of 63.2 ± 12.4 years. The most common reason for ICU admission was sepsis (n = 940, 63.2%). During their ICU stay, 29.7% of patients required vasoactive drug support (n = 442), 39.8% needed invasive mechanical ventilation support (n = 592), and 82 patients (5.5%) received renal replacement therapy. We conducted a multivariate COX proportional hazards model analysis, which revealed that BMI and a history of hypertension were protective factors. On the other hand, antitumor treatment within the 3 months prior to admission, transfer from the emergency department, general ward, or external hospital, high APACHE score, diagnosis of shock and respiratory failure, receiving invasive ventilation, and experiencing acute kidney injury (AKI) were identified as risk factors for poor prognosis within 90 days after ICU admission. The average length of stay in the ICU was 4 days, while the hospital stay duration was 18 days. A total of 415 patients died within 90 days after ICU admission, resulting in a mortality rate of 27.9%. We selected 8 indicators to construct the predictive model, which demonstrated good discrimination and calibration. The prognosis of cancer patients who are unplanned transferred to the ICU is generally poor. Assessing the risk factors and developing a risk prediction model for these patients can play a significant role in evaluating their prognosis.

## Introduction

In recent years, the survival rate of cancer patients has increased due to advancements in screening, detection, specific treatment, and side effect management. However, serious adverse events (SAEs) still occur frequently^1–4^. Unplanned ICU transfers refer to patients who are unexpectedly admitted to the intensive care unit from a lower level of care in the hospital^5^. It is evident that unplanned transfers to the ICU are associated with poorer outcomes and higher mortality rates^6–9^. Research indicates that the mortality rate for patients transferred to the ICU following elective surgery is approximately 11%. However, for patients transferred to the ICU after emergency surgery, the mortality rate rises to 37%, and those transferred from medical wards have an even worse in-hospital mortality rate of 58%^10^.

Various improvements have been made in the diagnosis and treatment of critically ill cancer patients. A 2016 article in the CA Cancer J Clin journal discussed and defined criteria for transferring critically ill cancer patients to the ICU, providing valuable guidance for their treatment^11^. Furthermore, observational studies suggest that early identification of changes in a patient's condition is crucial. Taking early measures can prevent eventual ICU admission, and if admitted, timely interventions can enhance clinical outcomes^12^. Additionally, Medical Emergency Teams (MET) have been found to play a significant role^13–15^. However, several questions remain unanswered, including the prognosis of patients with unplanned ICU admissions, the potential benefits of ICU admission, factors influencing prognosis, early assessment of a patient's condition, and how to evaluate the endpoint of empirical ICU treatments. These questions necessitate further exploration. Professor Elie Azoulay has highlighted the inadequacy of traditional mortality prediction indicators such as age, leukopenia, and malignant tumor characteristics, emphasizing the need for the development of new diagnostic tests^16^.

In light of this, our study aimed to analyze the risk factors associated with poor prognosis within 90 days of unplanned ICU admission. We have also developed a risk prediction model that aims to assist clinicians in better understanding the prognosis of their patients.

## Methods and materials

### Participating hospitals

ICU of 37 cancer hospitals in China.

### Patients

This retrospective multicenter cohort study focused on cancer patients admitted to the ICU of 37 cancer hospitals in China between May 2021 and July 2021. The study screened patients who were not initially scheduled for ICU admission.

### Inclusion criteria

Inclusion criteria for the study were patients who experienced unplanned ICU admission during the study period. Unplanned admission encompassed cases where patients were accidentally transferred to the ICU from a lower level of care in the hospital, including postoperative patients who were not initially scheduled for ICU transfer before anesthesia, emergency admissions, and patients who were unintentionally transferred to the ICU from general wards^17^.

### Data collection

Data collection involved gathering clinical information from a total of 1488 ICU patients. This included age, gender, height, weight, type of malignant tumor, and chronic underlying conditions such as hypertension, diabetes, coronary heart disease, COPD, chronic renal insufficiency, autoimmune diseases, chronic cardiac insufficiency, chronic hepatic insufficiency, and chronic respiratory insufficiency. Additionally, the treatment status within 3 months before ICU admission was recorded, including chemotherapy, radiotherapy, targeted therapy, immune checkpoint inhibitor therapy, and combination therapy. Acute illness severity was assessed within 24 h of initial ICU admission using the Acute Physiology and Chronic Health Assessment II (APACHE II) and the Systematic Sequential Organ Failure Score (SOFA) system.

For each patient, the following types and numbers of organ failure were recorded within 7 days of ICU admission : (i) Acute respiratory failure, defined as PaO_2_/FiO_2_ < 300 mmHg, respiratory rate > 25 breaths per minute, and symptoms of respiratory distress; (ii) shock; (iii) Acute kidney injury, as defined by the KIDGO guidelines, includes: ① an increase in serum creatinine (SCr) by 0.3 mg/dl (≥ 26.5 μmol/L) within 48 h; ② known or presumed renal damage occurring within 7 days, resulting in an SCr increase to more than 1.5 times the baseline value; ③ urine output less than 0.5 ml/(kg·h) sustained for 6 h; and (iv) sepsis is defined as a dysregulated response of the body to an infection causing life-threatening organ dysfunction. Sepsis 3.0 = infection + SOFA ≥ 2. Primary management types implemented in the ICU include conventional mechanical ventilation, vasopressors, and continuous renal replacement therapy (CRRT).

### Outcome measures

Length of ICU stay and length of hospital stay: Length of ICU or hospital stay was measured as the number of days from ICU admission to ICU discharge or hospital discharge. ICU mortality, in-hospital mortality, and antitumor treatment after ICU transfer were the final outcome of survival status 90 days after ICU transfer, and were divided into two groups: survivors 90 days after ICU admission and patients who died 90 days after ICU admission. Survival analysis of different survival status was conducted to find the risk factors affecting survival status.

### Statistical methods

The quantitative data were analyzed using t-tests for groups that followed a normal distribution, and Wilcoxon rank sum tests were used for groups with quantitative data that did not follow a normal distribution. Qualitative data were described using the number of cases and constituent ratios, and tests were used for group comparisons. Hazard ratios (HR) for each index were calculated using univariate COX regression models. Variables that showed statistically significant differences in univariate analysis (P < 0.05) and variables clinically relevant to patient prognosis were included in the multivariate COX regression model analysis. Multiple factor analysis was employed to select indicators with statistical significance for predicting model factors. The Best Subset Regression, combined with clinical significance, was used for screening and further construction of a nomogram to predict the 90-day prognosis.

### Ethics approval and consent to participate

This study conducted in accordance with the Declaration of Helsinki. Written informed Consent was obtained from all participants. The study was approved by Medical Ethics Committee of Tianjin Cancer Hospital. No. bc2021065, Apr.14, 2021.

## Results

**Figure 1:**
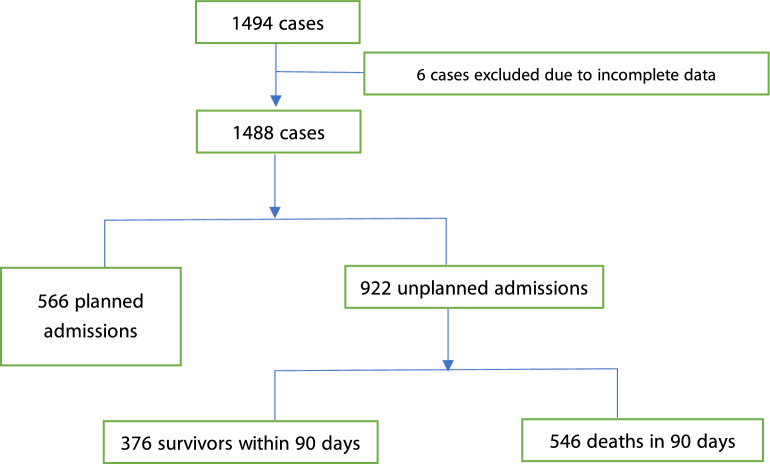
Flow diagram of the case collection.

Of the 37 intensive care units (ICUs) in cancer hospitals, 4 were excluded due to their busy clinical work, which affected case collection. A total of 1494 patients were included in the study, with 6 patients excluded for not meeting admission criteria or lack of information. The study included a total of 1488 patients from 33 ICUs in 26 provinces and cities, out of which 922 patients were selected for unplanned transfer. The flow chart depicting the patient selection process is shown in Fig. 1.

The 1488 cases included in the study were divided into a planned transfer group and an unplanned transfer group. The demographic and clinical characteristics of the patients are presented in Table 1. The unplanned transfer group consisted of 922 patients, with 574 males (62.3%) and 348 females (37.7%). The age of patients ranged from 9 to 90 years, with a mean age of 62.2 ± 12.2. The most common comorbidity among these patients was hypertension, present in 264 cases (28.6%). The majority of patients were transferred from general wards (79.8%). The top four tumor types among the included patients were esophageal cancer, lung cancer, abdominal tumors, and gynecological tumors. The primary reason for ICU admission was sepsis (n = 747, 81%), followed by respiratory failure (n = 468, 50.8%). Other complications observed among the patients included shock (n = 336, 36.4%), grade IV bone marrow suppression (n = 166, 18%), and acute kidney injury (n = 158, 17.1%). During ICU hospitalization, 38% of patients required vasopressor support (n = 352), 42.8% needed invasive mechanical ventilation support (n = 395), and 77 patients (8.3%) received renal replacement therapy.Clinical outcome: In the unplanned transfer patients, 84 (9.1%) died in ICU;102 cases (11.1%) died during hospitalization.376 patients died 90 days after ICU admission, with a mortality rate of 40.8%.In terms of the cause of death, 219 cases (58.2%) were related to tumor progression, and 157 cases (41.8%) were not related to tumor progression.After the transfer, 249 cases received antitumor therapy, accounting for 27.0%.

**Table 1:** Patients’ characteristics and outcomes according to planned ICU admission or not.

Variables	Total (n = 1488)	Planned ICU admission (n = 566)	Unplanned ICU admission (n = 922)	p
Type of cancer, n (%)				< 0.001
Esophageal cancer	607 (40.8)	272 (48.1)	335 (36.3)	
Lung cancer	285 (19.2)	79 (14)	206 (22.3)	
Abdominal cavity tumor	211 (14.2)	82 (14.5)	129 (14)	
Gynecological tumor	121 ( 8.1)	35 (6.2)	86 (9.3)	
Age, mean ± SD	63.2 ± 12.4	64.9 ± 12.6	62.2 ± 12.2	< 0.001
Sex, n (%)				0.364
Male	575 (38.6)	227 (40.1)	348 (37.7)	
Female	913 (61.4)	339 (59.9)	574 (62.3)	
BMI, mean ± SD	22.6 ± 3.7	23.3 ± 3.8	22.1 ± 3.7	< 0.001
Basic diseases				
Hypertension, n (%)				< 0.001
No	1015 (68.2)	357 (63.1)	658 (71.4)	
Yes	473 (31.8)	209 (36.9)	264 (28.6)	
Chronic respiratory insufficiency, n (%)				0.061
No	1463 (98.3)	561 (99.1)	902 (97.8)	
Yes	25 ( 1.7)	5 (0.9)	20 (2.2)	
Chronic hepatic insufficiency, n (%)				0.071
No	1467 (98.6)	562 (99.3)	905 (98.2)	
Yes	21 ( 1.4)	4 (0.7)	17 (1.8)	
Chemotherapy, n (%)				< 0.001
No	1108 (74.5)	491 (86.7)	617 (66.9)	
Yes	380 (25.5)	75 (13.3)	305 (33.1)	
Radiotherapy, n (%)				< 0.001
No	1401 (94.2)	557 (98.4)	844 (91.5)	
Yes	87 (5.8)	9 (1.6)	78 (8.5)	
Source of transfer, n (%)				< 0.001
Operating room	601 (40.4)	490 (86.6)	111 (12)	
Emergency room	65 (4.4)	1 (0.2)	64 (6.9)	
General ward	805 (54.1)	69 (12.2)	736 (79.8)	
Other hospital	17 (1.1)	6 (1.1)	11 (1.2)	
Surger or not				< 0.001
No	549 (36.9)	14 (2.5)	535 (58)	
Yes	939 (63.1)	552 (97.5)	387 (42)	
Organ function and support, n (%)				
APACHE II, Median (IQR)	12.0 (8.0, 17.0)	9.0 (6.0, 11.8)	15.0 (11.0, 20.0)	< 0.001
SOFA, median (IQR)	3.0 (2.0, 6.0)	2.0 (1.0, 3.0)	4.0 (3.0, 7.0)	< 0.001
Leukocyte grading				< 0.001
3.0-normal	1387 (93.2)	558 (98.6)	829 (89.9)	
2.0–3.0	26 (1.7)	3 (0.5)	23 (2.5)	
1.0–2.0	25 (1.7)	2 (0.4)	23 (2.5)	
< 1.0	49 (3.3)	2 (0.4)	47 (5.1)	
Granulocyte grading				< 0.001
1.5-normal	1394 (93.7)	558 (98.6)	836 (90.7)	
1.0–1.5	18 (1.2)	3 (0.5)	15 (1.6)	
0.5–1.0	15 (1.0)	0 (0)	15 (1.6)	
< 0.5	60 (4.0)	4 (0.7)	56 (6.1)	
Grade four myelosuppression				< 0.001
No	1297 (87.2)	541 (95.6)	756 (82)	
Yes	191 (12.8)	25 (4.4)	166 (18)	
Sepsis				< 0.001
No	548 (36.8)	373 (65.9)	175 (19)	
Yes	940 (63.2)	193 (34.1)	747 (81)	
Respiratory failure				< 0.001
No	922 (62.0)	468 (82.7)	454 (49.2)	
Yes	566 (38.0)	98 (17.3)	468 (50.8)	
Conventional oxygen therapy				< 0.001
No	234 (15.7)	32 (5.7)	202 (21.9)	
Yes	1254 (84.3)	534 (94.3)	720 (78.1)	
High flow oxygen therapy				< 0.001
No	1131 (76.0)	487 (86)	644 (69.8)	
Yes	357 (24.0)	79 (14)	278 (30.2)	
Noninvasive ventilation				0.004
No	1429 (96.0)	554 (97.9)	875 (94.9)	
Yes	59 ( 4.0)	12 (2.1)	47 (5.1)	
Invasive ventilation				0.002
No	896 (60.2)	369 (65.2)	527 (57.2)	
Yes	592 (39.8)	197 (34.8)	395 (42.8)	
Shock				< 0.001
No	1076 (72.3)	490 (86.6)	586 (63.6)	
Yes	412 (27.7)	76 (13.4)	336 (36.4)	
Use of vasoactive drugs				< 0.001
No	1046 (70.3)	474 (83.7)	572 (62)	
Yes	442 (29.7)	92 (16.3)	350 (38)	
AKI				< 0.001
No	1313 (88.2)	549 (97)	764 (82.9)	
Yes	175 (11.8)	17 (3)	158 (17.1)	
CRRT				< 0.001
No	1406 (94.5)	561 (99.1)	845 (91.6)	
Yes	82 ( 5.5)	5 (0.9)	77 (8.4)	
Clinical outcome				
ICU stay, median (IQR)	4.0 (2.0, 7.0)	3.0 (2.0, 6.0)	5.0 (3.0, 8.0)	< 0.001
Hospital stay, median (IQR)	18.0 (12.0, 28.0)	18.0 (12.0, 26.0)	19.0 (12.0, 28.0)	0.057
ICU motality, n (%)				< 0.001
No	1400 (94.1)	562 (99.3)	838 (90.9)	
Yes	88 ( 5.9)	4 (0.7)	84 (9.1)	
Hospital motality, n (%)				< 0.001
No	1380 (92.7)	560 (98.9)	820 (88.9)	
Yes	108 ( 7.3)	6 (1.1)	102 (11.1)	
Anti-tumor therapy after transfer out, n (%)				< 0.001
No	1000 (67.2)	327 (57.8)	673 (73)	
Yes	488 (32.8)	239 (42.2)	249 (27)	
Tumor progression-related death,n(%)				< 0.001
NO	1252 (84.1)	549 (97)	703 (76.2)	
YES	236 (15.9)	17 (3)	219 (23.8)	
Non-tumor progression-related death, n (%)				< 0.001
No	1309 (88.0)	544 (96.1)	765 (83)	
Yes	179 (12.0)	22 (3.9)	157 (17)	
Survival 90 days, n (%)				< 0.001
Yes	1073 (72.1)	527 (93.1)	546 (59.2)	
No	415 (27.9)	39 (6.9)	376 (40.8)	

We conducted univariate and multivariate analysis based on 90-day mortality groups. Univariate analysis showed (see Table 2) that lung cancer,femininity, high BMI, and history of hypertension are protective factors for death within 90 days of admission (hindering the occurrence of death), a history of chronic cardiac insufficiency, antitumor therapy 3 months before admission, transfer from the emergency department, general ward and external hospital, fourth-degree bone marrow suppression, high APACHE score, high SOFA score, sepsis, diagnosis of shock, use of vasoactive drugs, respiratory failure, receiving invasive ventilation, AKI, and renal replacement therapy are risk factors for poor prognosis (accelerating death) within 90 days after ICU admission. On the basis of univariate analysis, a multivariate COX proportional risk model was established (see Table 3). It shows that BMI and hypertension history were protective factors, while antitumor treatment 3 months before admission, transfer from the emergency department, general ward and external hospital, high APACHE score, shock diagnosis and respiratory failure. receiving invasive ventilation and AKI were risk factors for poor prognosis within 90 days after ICU admission.

**Table 2:** Univariate analysis of prognosis within 90 days of unplanned ICU admissions.

Influencing factors	Univariate analysis		
	HR	95%CI	P
Type of tumor:ref. = 1			
Lung cancer	2.67	1.4–5.08	
Gender (female vs. male)	0.785	0.634–0.973	0.027
BMI (per 1 kg/m^2^ increase)	0.924	0.897–0.951	< 0.001
History of hypertension (yes vs. no)	0.732	0.577–0.927	0.010
History of chronic cardiac insufficiency (yes vs. no)	1.841	1.035–3.273	0.038
Source of transfer (emergency vs operating room)	4.286	2.298–7.996	< 0.001
Source of transfer (general ward vs operating room)	4.090	2.437–6.896	< 0.001
Source of transfer (other hospital vs operating room)	5.413	2.100–13.955	< 0.001
Surgery or not (yes vs no)	0.33	0.26–0.41	< 0.001
Grade 4 myelosuppression (yes vs. no)	1.420	1.110–1.815	0.005
APACHE score (for each point increase)	1.060	1.048–1.072	< 0.001
SOFA score (for each point increase)	1.119	1.092–1.146	< 0.001
Sepsis (yes vs. no)	2.405	1.720–3.364	< 0.001
Diagnosis of shock (yes vs. no)	2.042	1.668–2.501	< 0.001
Use of vasoactive (yes vs. no)	1.877	1.533–2.299	< 0.001
Respiratory failure (yes vs. no)	2.312	1.869–2.861	< 0.001
Invasive ventilation (received vs. not received)	2.054	1.675–2.518	< 0.001
AKI (yes vs. no)	2.443	1.948–3.065	< 0.001
Renal replacement therapy (received vs. not received)	2.058	1.528–2.773	< 0.001

**Table 3:** Multivariate analysis of prognosis within 90 days of unplanned ICU admissions.

Influencing factors	Multivariate analysis		
	HR	95%CI	P
Gender (female vs. male)	0.916	0.734–1.143	0.437
BMI (per 1 kg/m^2^ increase)	0.945	0.916–0.974	< 0.001
History of hypertension (yes vs. no)	0.768	0.602–0.980	**0.034**
History of chronic cardiac insufficiency (yes vs. no)	1.132	0.628–2.043	0.680
Surgery or not (yes vs. no)	0.35	0.27–0.44	** < 0.001**
Source of transfer (emergency vs operating room)	3.910	2.059–7.426	** < 0.001**
Source of transfer (general ward vs operating room)	4.162	2.458–7.047	** < 0.001**
Source of transfer (other hospital vs operating room)	4.964	1.870–13.176	**0.001**
Grade 4 myelosuppression (yes vs. no)	1.016	0.771–1.338	0.912
APACHE score (for each point increase)	1.024	1.008–1.041	**0.004**
SOFA score (for each point increase)	1.008	0.974–1.043	0.651
Sepsis (yes vs. no)	1.300	0.909–1.860	0.151
Diagnosis of shock (yes vs. no)	2.017	1.206–2.501	**0.007**
Use of vasoactive drug(yes vs. no)	0.601	0.359–1.006	0.053
Respiratory failure (yes vs. no)	1.544	1.189–2.005	**0.001**
Invasive ventilation (received vs. not received)	1.620	1.243–2.112	** < 0.001**
AKI (yes vs. no)	1.698	1.241–2.324	**0.001**
Renal replacement therapy (received vs. not received)	0.921	0.626–1.355	0.677

Significant values are in bold.

### Establish a prediction model

#### Model development

**Figure 2:**
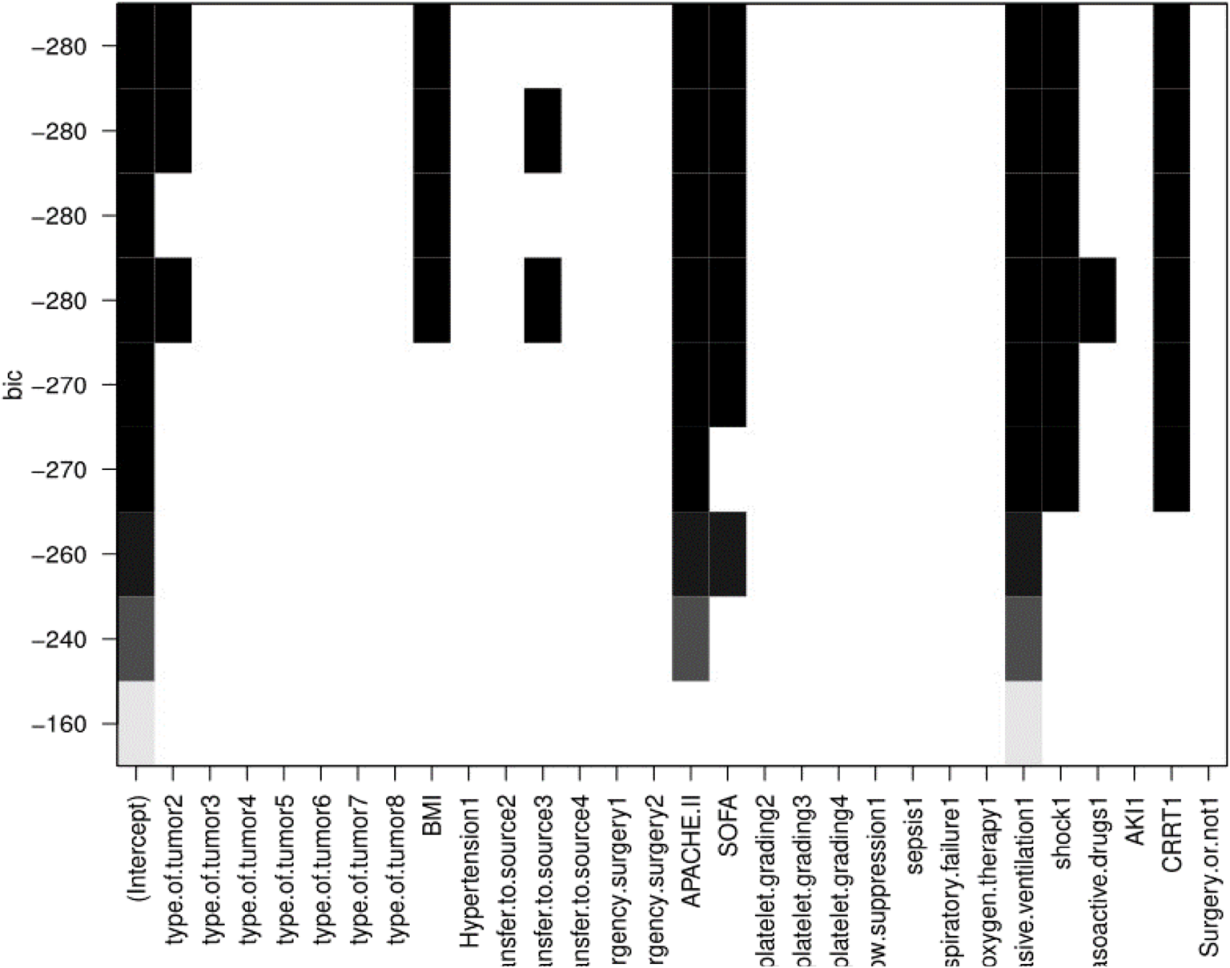
Variables selection using the BSR.

**Figure 3:**
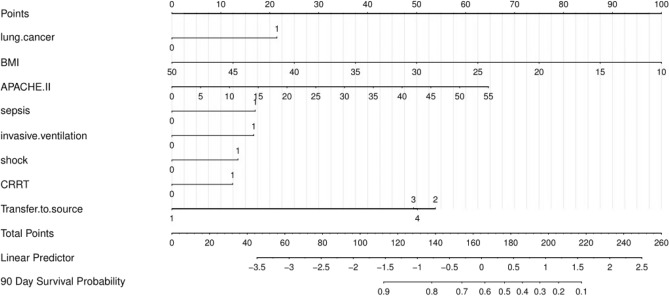
Nomogram of the 90-day prognosis of patients with unplanned ICU transfer.

A multi-factor analysis was conducted to consider twelve risk factors. The Best Subsets Regression (BSR) method was used to select seven significant indicators for the predictive model(Fig. 2), referred to as Model 1. The indicators of Model 1 include type of tumor2 (lung cancer), BMI, APACHE II, SOFA,invasive ventilation, shock, CRRT. Based on clinical relevance and consideration, the SOFA score was removed and sepsis and source of transfer were added, resulting in Model 2. The indicators of Model 2 are type of tumor2 (lung cancer), BMI, APACHE II, invasive ventilation, shock, CRRT, sepsis, and source of transfer. The predictive performance of the two models was evaluated by comparing the Area Under the Curve (AUC) of their ROC curves. Model 2 exhibited a higher AUC (77.07) compared to Model 1 (74.3). Finally, a nomogram was plotted to predict the 90-day prognosis of patients with unplanned ICU transfer based on the model, as shown in Fig. 3.

#### Model evaluation

**Figure 4:**
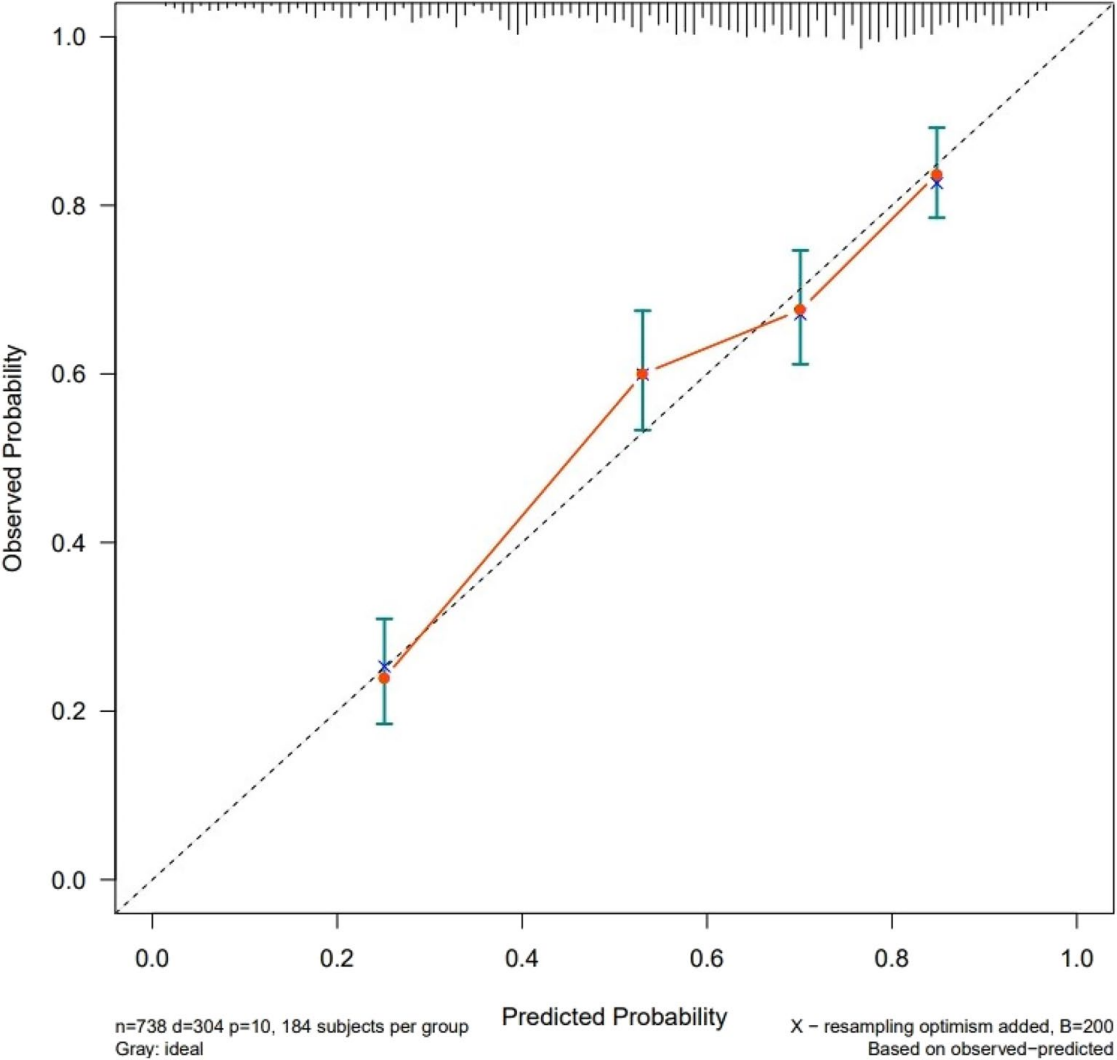
Probability calibration of model-predicted 90-day survival versus actual survival in patients with unplanned ICU transfer.

The predictive ability of the model was assessed in terms of discrimination (measured by C-index) and calibration. The C-index was found to be 0.772, indicating a reasonably accurate predictive ability. The calibration curve in Fig. 4 demonstrates good agreement between the predicted risk and the actual outcomes. The curve closely aligns with the 45-degree reference line, indicating a well-calibrated model.

To determine the 90-day survival probability of a patient, follow these steps: First, locate the individual's admission profile on the appropriate axis. Using a pencil and ruler, draw a line vertically up to the top 'Points' axis. Next, sum the two points to create a 'Total Points' score. Finally, draw a line vertically down from the 'Total Points' axis through the '90 Day Survival Probability' axis to obtain the future survival probability. For instance, consider a patient with lung cancer who was admitted to the ICU from the emergency department due to shock. During their time in the ICU, they required invasive ventilation and CRRT. Based on the calculations, the patient's 90-day survival probability is less than 10% (total points: 308; lung cancer: 56 points, emergency department: 140 points, shock: 36 points, invasive ventilation: 44 points, CRRT: 32 points).

## Discussion

According to an analysis of the National Audit and Research Centre for Intensive Care (ICNARC) database in the UK, it was discovered that patients with severe tumors often experience physiological disorders a few hours or even a few days before their condition worsens. In 2012, there were approximately 40,000 unplanned admissions to the ICU, with up to 80% of these patients experiencing pre-clinical deterioration^18–20^. Our study found that out of 922 patients who were transferred to the ICU unexpectedly, the ICU mortality rate was 9.1%. The in-hospital mortality rate was 11.1%, with 376 patients dying 90 days after ICU admission, resulting in a mortality rate of 40.8%. These findings align with data from other studies conducted abroad^21–23^. Further subgroup analysis revealed that out of 736 patients transferred through the general ward, the in-hospital mortality rate was 12.2% and the 90-day mortality rate was 35.3%. The mortality rates of patients transferred through the general ward were significantly higher compared to those transferred through the operating room (0.9% and 1.6% respectively). A prospective, multicenter cohort study conducted in 28 Brazilian ICUs found that the in-hospital mortality rate was 58% among patients admitted to the ICU due to an unexpected event in the general ward, compared to 37% and 11% among patients admitted to the ICU after surgery, including emergency or planned surgery^24^. In that same study, the ICU mortality rate was 6% among patients admitted after elective surgery, compared to 23% among patients admitted after emergency surgery^24^. Soares et al. also investigated the mortality rates among cancer patients admitted for unplanned surgery and found ICU and in-hospital mortality rates of 23% and 37% respectively. Patients with medical cancer had even higher mortality rates, with ICU and hospital mortality rates of 44% and 58% respectively^24^. This study indicates that unplanned admissions to the ICU have a significantly higher mortality rate compared to planned admissions. Among these patients, those transferred from the ICU to the general ward have a higher mortality rate than patients transferred directly to the ICU through the operating room. The possible reason for this could be the failure to promptly recognize changes in patients' condition, resulting in delayed ICU treatment. As a result, these patients are admitted to the ICU in a critical condition, sometimes even reaching an irreversible state.

In order to improve the prognosis of patients with unplanned transfer and avoid unnecessary suffering for those with irreversible disease, it is necessary to better understand the epidemiology and identify the prognostic factors specific to this patient population^25^. Previous studies have mainly focused on identifying clinical variables associated with poor ICU outcomes, but there have been limited studies on populations with unplanned ICU transfers, and even fewer studies on patients with severe cancer^26–29^. One notable study conducted by James Malycha et al.^30^ retrospectively analyzed 16 studies to identify factors associated with unplanned ICU admission. They found that two comorbidities (congestive heart failure and diabetes), two demographic characteristics (advancing age and male sex), one diagnosis (liver disease), and six vital signs (respiratory rate, heart rate, body temperature, systolic and diastolic blood pressure, and arterial oxygen saturation) had strong associations with unplanned ICU admission. The passage also discusses the use of early warning scores, which incorporate vital signs, and the establishment of specialized rapid response teams/medical emergency teams to promptly detect changes in patient condition and provide appropriate interventions. The MERIT study, a multicenter randomized controlled trial, demonstrated a significant decrease in adverse outcomes with an increase in the response of medical emergency teams^31^. Another study in Japan examined the relationship between hospital capacity, RRS (Rapid Response System) call rates, and clinical outcomes of activated RRS patients, concluding that higher RRS call rates in hospitals resulted in a decrease in unplanned ICU admissions^32^. Similar studies have also shown a strong correlation between changes in vital signs and unplanned ICU admissions^14,33,34^. In our study, we focused on analyzing organ damage and organ support rather than redundant statistical analyses. It is crucial to examine the deteriorating patient conditions and the functional status of organs upon ICU admission, as they provide critical insights for subsequent treatment. We used the 90-day survival status as the final outcome, dividing it into two groups: the 90-day survival group and the 90-day death group. We analyzed the risk factors of both groups to identify indicators that could impact patient prognosis. Multivariate analysis revealed several predictive factors for poor prognosis of UIA. These included high BMI and a history of hypertension as protective factors, antitumor therapy 3 months before admission, transfer to the emergency department, general ward, or other hospitals, high APACHEII score, and organ injury (such as shock, respiratory failure, and AKI). Organ support, specifically receiving invasive ventilation, was identified as a risk factor for poor prognosis within 90 days after admission to UIA. Studies have shown that patients with overweight or obesity (excluding morbid obesity) have a 60-day reduction in mortality compared to those with normal BMI^35^, This may be attributed to the increased activity of the renin-angiotensin system^36^ and the ability of lipoproteins and adipocytes to inhibit the release of harmful inflammatory mediators^37,38^. When organ dysfunction such as shock occurs, individuals with a history of hypertension are more likely to maintain a higher blood pressure level, which can be considered a favorable factor for short-term prognosis. However, further studies are needed to explore the mechanisms behind this. On the other hand, we observed that patients with a history of tumor treatment within 3 months prior to admission had a poor prognosis, possibly due to varying degrees of organ damage during the treatment. Additionally, our analysis revealed that patients admitted from general wards had a worse prognosis compared to those from operating rooms. Statistical results indicated that a high APACHE II score was an independent risk factor for poor prognosis, while the SOFA score did not demonstrate comparable predictive power to the APACHE II score, which is consistent with the findings of previous studies^39,40^. Among the various organ injuries, acute respiratory failure was found to be the primary reason for ICU admission. A secondary analysis of the EFRAIM study by Soraya Benguerfi et al. revealed that the main causes of acute respiratory failure among cancer patients admitted to the ICU included infection, non-pulmonary sepsis, cancer-related acute lung injury, and compensation for chronic diseases^41^. It is worth noting that acute respiratory failure often necessitates mechanical ventilation^29^, which, compared to other oxygen therapies, is a major negative prognostic factor and is associated with a significant increase in mortality^42,43^. The presence of shock was a significant factor leading to unplanned transfers to the ICU, with an incidence rate of 36.4%. Septic shock was found to be the most common type of shock. Additionally, acute renal failure was identified as a predictor of poor prognosis. It occurs in 12% to 36% of cancer patients and is associated with high morbidity and mortality^44,45^. Studies have indicated that 16% to 23% of cancer patients admitted to the ICU develop severe renal failure and require renal replacement therapy^46,47^. Our findings revealed that the incidence of acute kidney injury (AKI) was 17.1%, and further continuous renal replacement therapy (CRRT) support was necessary in 8.3% of cases. The etiology of renal failure in cancer patients is often multifactorial and may be attributed to the cancer itself, cancer treatment, or related complications^48,49^.

This study aimed to analyze the clinical factors associated with poor prognosis in unplanned ICU transfers. The findings can help identify high-risk groups, particularly those who were empirically admitted to the ICU, to some extent, and avoid unnecessary overtreatment and patient suffering. The evolution of organ dysfunction following intensive ICU treatment may serve as a more reliable predictor of prognosis compared to various scores assessed prior to admission^50^. Instead of making decisions about providing intensive care based on static parameters assessed at admission, the decision to continue full intensive care should be based on the patient's changing condition. While the use of nomograms to predict individual patient risks has been widely reported in cancer research, the prognostic risk of unplanned referrals in patients with severe tumors is rarely studied. However, it is important not to ignore this population in cancer treatment as it is crucial for determining the subsequent treatment direction. To address this, we have developed a risk prediction model based on multiple factors analysis and mapped it to a nomogram. This nomogram can help physicians evaluate the prognosis of patients receiving empirical therapy in the ICU and assist in determining the next steps for treatment. It represents an important innovation in ICU patient prognosis assessment^51^.

Our study also has some limitations. Firstly, the duration of the study was short, with only 2 months of clinical data collected. A longer prospective study would be needed to account for any time-dependent effects on disease incidence. Secondly, the evaluation indicators used were not comprehensive enough. For instance, due to the low incidence of neurological severe disease in cancer hospitals, the population of patients who were unplanned transferred due to neurological severe disease was not statistically analyzed. Furthermore, the accuracy of the linear map needs to be verified with more clinical data before it can be widely adopted.

## Conclusions

This study is the first multicenter study in China to focus on severe cancer patients who are not scheduled to be transferred to the ICU. We have described the general characteristics of this population and proposed prognostic factors. Additionally, we have developed a risk prediction model that can guide treatment and prognosis in the ICU for this population.

## Data Availability

The data that support the findings of this study are available from Cancer Critical Care Committee of China Anti-Cancer Association but restrictions apply to the availability of these data, which were used under license for the current study, and so are not publicly available. Data are however available from the authors upon reasonable request and with permission of Cancer Critical Care Committee of China Anti-Cancer Association.
